# Knockdown of the *Drosophila* Fused in Sarcoma (FUS) Homologue Causes Deficient Locomotive Behavior and Shortening of Motoneuron Terminal Branches

**DOI:** 10.1371/journal.pone.0039483

**Published:** 2012-06-19

**Authors:** Hiroshi Sasayama, Mai Shimamura, Takahiko Tokuda, Yumiko Azuma, Tomokatsu Yoshida, Toshiki Mizuno, Masanori Nakagawa, Nobuhiro Fujikake, Yoshitaka Nagai, Masamitsu Yamaguchi

**Affiliations:** 1 Department of Neurology, Graduate School of Medical Science, Kyoto Prefectural University of Medicine, Kamigyo-ku, Kyoto, Japan; 2 Department of Applied Biology, Kyoto Institute of Technology, Matsugasaki, Sakyo-ku, Kyoto, Japan; 3 Insect Biomedical Research Center, Kyoto Institute of Technology, Matsugasaki, Sakyo-ku, Kyoto, Japan; 4 Department of Molecular Pathobiology of Brain Diseases, Graduate School of Medical Science, Kyoto Prefectural University of Medicine, Kamigyo-ku, Kyoto, Japan; 5 Department of Degenerative Neurological Diseases, National Institute of Neuroscience, National Center of Neurology and Psychiatry, Kodaira, Tokyo, Japan; Thomas Jefferson University, United States of America

## Abstract

Mutations in the fused in sarcoma/translated in liposarcoma gene (FUS/TLS, FUS) have been identified in sporadic and familial forms of amyotrophic lateral sclerosis (ALS). FUS is an RNA-binding protein that is normally localized in the nucleus, but is mislocalized to the cytoplasm in ALS, and comprises cytoplasmic inclusions in ALS-affected areas. However, it is still unknown whether the neurodegeneration that occurs in ALS is caused by the loss of FUS nuclear function, or by the gain of toxic function due to cytoplasmic FUS aggregation. Cabeza (Caz) is a *Drosophila* orthologue of human FUS. Here, we generated *Drosophila* models with *Caz* knockdown, and investigated their phenotypes. In wild-type *Drosophila*, Caz was strongly expressed in the central nervous system of larvae and adults. Caz did not colocalize with a presynaptic marker, suggesting that Caz physiologically functions in neuronal cell bodies and/or their axons. Fly models with neuron-specific *Caz* knockdown exhibited reduced climbing ability in adulthood and anatomical defects in presynaptic terminals of motoneurons in third instar larvae. Our results demonstrated that decreased expression of *Drosophila* Caz is sufficient to cause degeneration of motoneurons and locomotive disability in the absence of abnormal cytoplasmic Caz aggregates, suggesting that the pathogenic mechanism underlying FUS-related ALS should be ascribed more to the loss of physiological FUS functions in the nucleus than to the toxicity of cytoplasmic FUS aggregates. Since the *Caz*-knockdown *Drosophila* model we presented recapitulates key features of human ALS, it would be a suitable animal model for the screening of genes and chemicals that might modify the pathogenic processes that lead to the degeneration of motoneurons in ALS.

## Introduction

Amyotrophic lateral sclerosis (ALS) is a devastating neurodegenerative disease that is characterized by degeneration of motor neurons, which leads to progressive muscle weakness and eventually fatal paralysis, typically within 1 to 5 years after disease onset [1]. Frontotemporal lobar degeneration (FTLD) is a clinically diverse dementia syndrome, with phenotypes that include behavioral changes, semantic dementia and progressive non-fluent aphasia [2]. Although these two diseases are clinically distinct and affect different parts of the central nervous system, it has been long thought that these two diseases are related since ALS patients often develop cognitive deficits with frontotemporal features and FTLD patients can present symptoms of motor neuron disease [3], [4]. This hypothesis, which was derived from clinical observations, has been biochemically confirmed by identification of the 43 kDa TAR-DNA-binding protein (TDP-43) as the major aggregating protein in subtypes of both ALS and FTLD (ALS-TDP and FTLD-TDP, respectively) [5], [6]. Moreover, over 30 different mutations in the TDP-43 gene (*TARDBP*) have been identified in various sporadic and familial ALS patients [7]–[12], and subsequently TDP-43 mutations were reported in various FTLD-TDP cases [13], [14]. Shortly after the identification of mutations in TDP-43 in ALS cases, mutations in another gene encoding an RNA-binding protein, FUS (fused in sarcoma; also known as TLS, translocated in liposarcoma), were identified in cases with familial ALS (ALS-FUS) [15], [16]. Both dominantly and recessively inherited *FUS* mutations have been reported in familial ALS [15], and *FUS* mutations may be more common than *TARDBP* mutations in familial ALS [17]. Additional mutations in *FUS* have recently been identified in sporadic ALS cases and in a subset of FTLD cases (FTLD-FUS) [18], [19]. FUS is normally a nuclear protein, but cytoplasmic FUS-immunoreactive inclusions were demonstrated in lower motor neurons of ALS patients harboring *FUS* mutations [16]. Cytoplasmic aggregation of wild-type FUS was subsequently reported as the prominent disease phenotype in other neurodegenerative diseases such as basophilic inclusion body disease [20], some types of juvenile ALS [21], and in the majority of tau- and TDP43-negative FTLD [22]. The identification of these two RNA-binding proteins that aggregate and are sometimes mutated in ALS and FTLD gave rise to the emerging concept that disturbances in RNA regulation may play a major role in the pathogenesis of ALS and FTLD [23]. Moreover, FUS aggregation is also demonstrated in Huntington's disease, spinocerebellar ataxia types 1, 2, and 3, and dentatorubropallidoluysian atrophy [24], [25]. These findings suggest an important role for FUS aggregation in the pathogenesis of neurodegenerative diseases beyond ALS and FTLD.

FUS is a ubiquitously expressed, 526 amino acid protein that was initially identified as a proto-oncogene, and which causes liposarcoma due to chromosomal translocation [26]. FUS is an RNA-binding protein that is implicated in multiple aspects of RNA metabolism including microRNA processing, RNA splicing, trafficking and translation [23], [27], [28]. FUS shows nuclear and cytoplasmic expression and shuttles between the nucleus and the cytoplasm [27], [29]. In neurons, FUS is localized to the nucleus but it is transported to dendritic spines at excitatory post-synapses in a complex with RNA and other RNA-binding proteins [30]. Similar to TDP-43, FUS comprises a glycine-rich domain (GRD), an RNA-recognition-motif (RRM) domain and a nuclear localization sequence (NLS). ALS/FTLD-associated mutations cluster in the C-terminal region of the FUS protein that contains a non-classical R/H/KX_2–5_PY NLS motif [31] as well as in the GRD motif that is important for protein-protein interactions and also exists in the C-terminal region of TDP-43. Most pathogenic mutations of the *TARDBP* gene cluster in this GRD motif. The only known genetic cause for ALS/FTLD with FUS pathology is mutations in the *FUS* gene itself. The *FUS* mutations in the NLS-containing C-terminal region lead to redistribution of the FUS protein from the nucleus to the cytoplasm [32]–[35]. These findings suggest that the loss of physiological nuclear functions of FUS that involve RNA regulation may contribute to the pathogenesis of ALS/FTLD.

There is a single homolog for each of human FUS and TDP-43 in *Drosophila*, named Cabeza (Caz) and TBPH, respectively. The *Caz* gene is located on the X chromosome, and is a member of an RNA binding proteins that are conserved from fly to man. *In situ* hybridization and immunohistochemical analyses demonstrated that Caz mRNA and protein are enriched in the brain and CNS during embryogenesis, and the Caz protein was detected in the nuclei of several larval tissues and in imaginal discs [36]. The full-length recombinant Caz protein and its RRM domain are capable of binding RNA in vitro [36]. These findings suggest that Caz is a nuclear RNA binding protein that may play an important role in the regulation of RNA metabolism during fly development. Feiguin et al. reported that *Drosophila* lacking TBPH presented deficient locomotive behaviors, reduced life span and anatomical defects at neuromuscular junctions (NMJ), suggesting that a loss of TDP-43 nuclear functions could be a causative factor of the neurodegeneration observed in patients with ALS/FTLD [37].

As mentioned above, the loss of the nuclear function of FUS or TDP-43 plays an important role in the pathogenesis of ALS/FTLD. However, aggregation of TDP-43 or FUS may by itself be toxic due to a toxic gain-of-function associated with the formation of cytoplasmic aggregates of those proteins, which would trap vital proteins and/or RNAs and might disturb cellular homeostasis. Thus, it remains unclear whether it is the loss of FUS nuclear function or the gain of toxic function resulting from FUS aggregation that is the mechanism that underlies the primary abnormality that leads to the neurodegeneration that occurs in ALS/FTLD. The existence of both dominantly and recessively inherited *FUS* mutations in familial ALS has provoked further controversy regarding whether the underlying pathogenic mechanism of ALS/FTLD is due to gain-of-toxic-function or loss-of-nuclear function [15], [19], [38]. Here, we investigated phenotypes of fly models with knockdown of the *Drosophila* FUS homologue, *Caz* gene, to provide supporting evidence for our hypothesis that the pathogenesis of ALS/FTLD may be due more to the loss of physiological FUS functions than to the toxicity of its cytoplasmic aggregates. Neuron-specific knockdown of the *Drosophila Caz* gene reduced the climbing abilities of adult flies as well as caused anatomical defects, such as a reduced length of synaptic branches, in presynaptic terminals of motoneurons in third instar larvae, suggesting that decreased expression of the *Drosophila* FUS homologue may be sufficient for development of the degeneration of motoneurons and for the deficient locomotive behavior in this model fly.

## Results

### Comparison of the amino acid sequence of human FUS and *Drosophila* Caz

**Figure 1:**
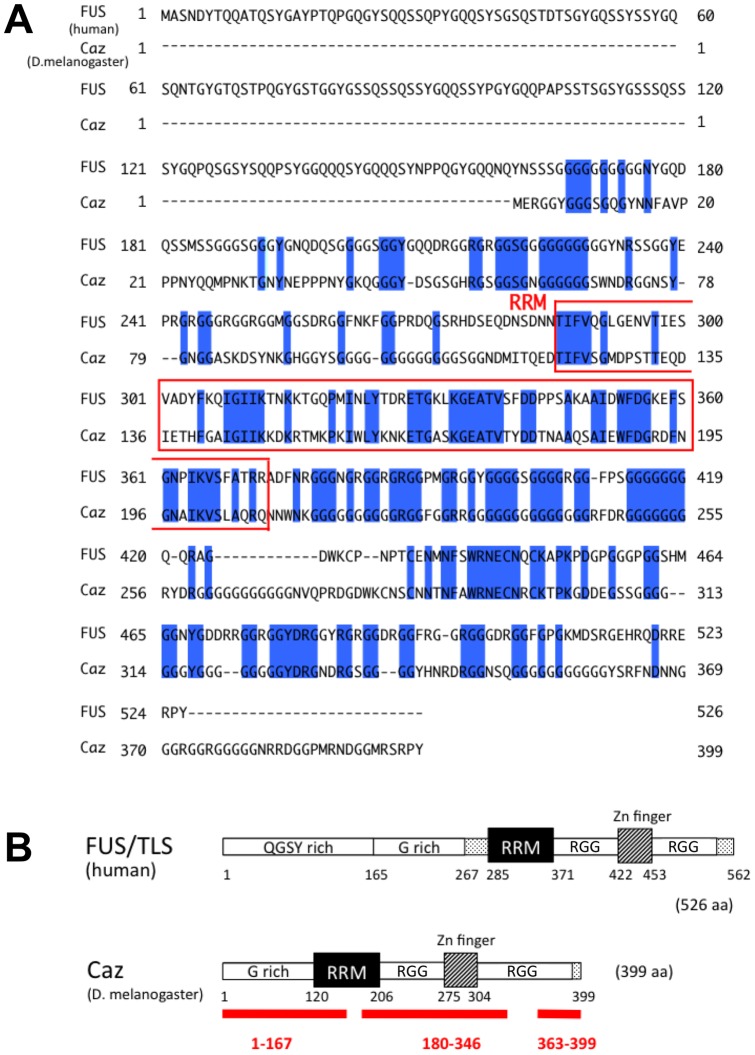
Comparison of human FUS and *Drosophila* Caz. (A) Alignment of human FUS and *Drosophila* Caz amino acid sequences. Identity is indicated in blue. The RNA-recognition-motif (RRM) domain is outlined with a red box. (B) Schematic drawings of domain structures of Human FUS and *Drosophila* Caz proteins. The human FUS protein contains an N-terminal QGSY-rich domain, which functions as a potent transcriptional activation domain [62]–[64]. The glycine-rich domain (G rich), RRM domain, a domain containing multiple Arg-Gly-Gly (RGG) motifs and a zinc finger (ZnF), are all involved in RNA binding [65], [66]. A solid line under the schema of *Drosophila* Caz shows the target genomic sequence of each of the three RNAi transgenes employed in this study, *UAS-Caz-IR_1_*_–*167*_, *UAS-Caz-IR_180_*_–*346*_ and *UAS-Caz-IR_363-399_*.

The amino acid sequence of *Drosophila* Caz was retrieved from the Flybase and was compared with that of human FUS using BLAST and FASTA (Figure 1). The identity and the similarity of the amino acid sequences of Caz and FUS are 44.9% and 62.3%, respectively. Regarding conservation of specific FUS domains, the RRM domain, which is known to bind RNAs, as well as the zinc finger domain, are both highly conserved between human FUS and *Drosophila* Caz, showing 50% and 63% identity, respectively. The similarity of the human and *Drosophila* RRM and zinc finger domains is as high as 75% and 73%, respectively.

### Specificity of the anti-Caz antibody

**Figure 2:**
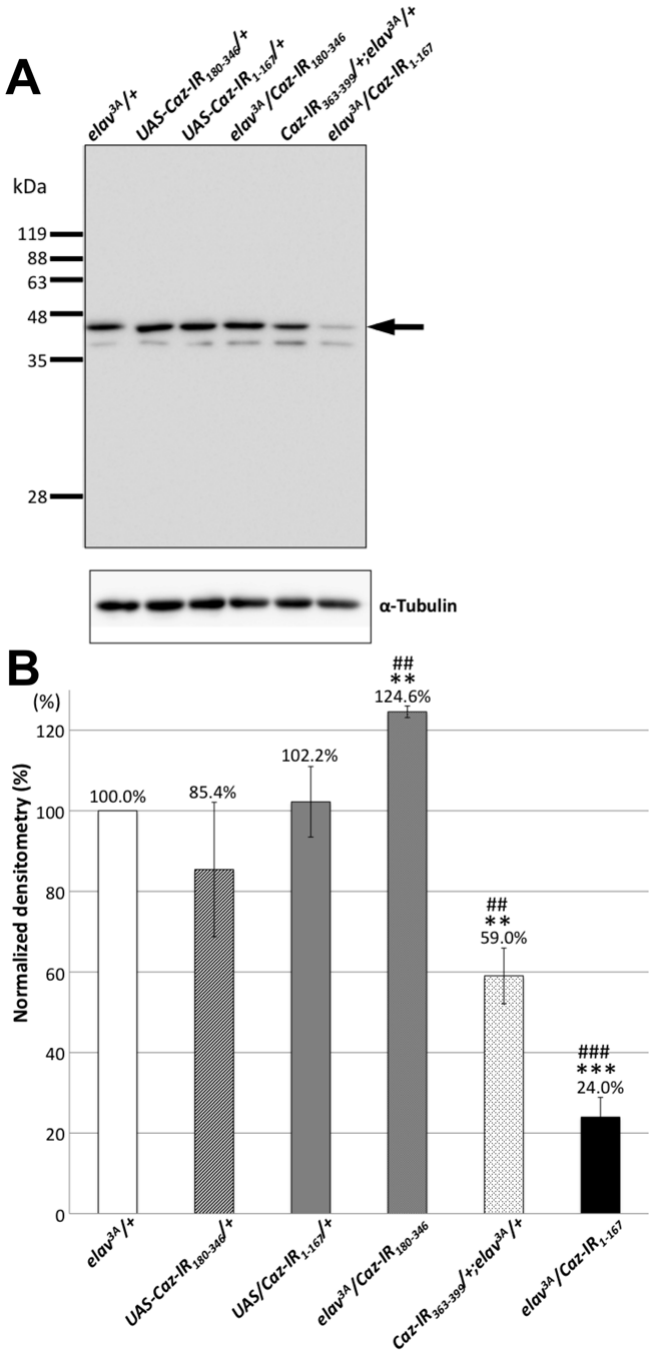
Western immunoblot analysis of the CNS extracts of third instar larvae. (A) A representative result of the analysis of protein extracts from the CNS of the driver control (*elav^3A^/+*) and responder control (*UAS-Caz-IR_180-346_/+* and *UAS-Caz-IR_1-167_/+*) flies (n = 5, each) and transgenic flies (*elav^3A^/Caz-IR_180-346_*, *Caz-IR_363-399_/+;elav^3A^/+* and *elav^3A^/Caz-IR_1-167_*) (n = 5, each). The blots were probed with the polyclonal anti-Caz antibody that was newly raised for this study. α-Tubulin was used as a loading control. A 45-kDa band (arrow) corresponds to the Caz protein. (B) Densitometric quantification of the 45-kDa bands derived from triplicated immunoblot analyses of the CNS tissues of each fly strain in (A). The intensity of the 45 kDa band which indicates the expression level of Caz protein was much weaker in flies carrying *elav^3A^/Caz-IR_1-167_* or *Caz-IR_363-399_/+;elav^3A^/+* than in the driver and responder control flies. The columns and horizontal bars indicate the mean values and the standard errors of the triplicated experiments. **p<0.01 (vs. *elav^3A^/+*), *** p<0.001 (vs. *elav^3A^/+*), ^##^p<0.01 (vs. *UAS-Caz-IR_1-167_/+*), ^###^p<0.001 (vs. *UAS-Caz-IR_1-167_/+*).

We raised a polyclonal antibody against a mixed peptide corresponding to residues 30–45 and 382–390 of *Drosophila* Caz for immunological studies. In order to confirm the specificity of this antibody, we used this anti-Caz polyclonal antibody for immunoblotting analyses of CNS extracts of third instar larvae carrying *elav^3A^-GAL4/+* (*elav^3A^/+*, a driver control fly), *UAS-Caz-IR/+* (a responder control fly), and RNAi transgenes encoding inverted repeats corresponding to various Caz regions, *elav^3A^-GAL4>UAS-Caz-IR* (Figure 2). A single major band with an apparent molecular weight of 45 kDa was detected on immunoblots of all of the flies using the anti-Caz antibody (Figure 2A). Although the size of this protein was slightly larger than the size (38.8 kDa) of the Caz protein predicted based on its amino acid composition, the intensity of this band was significantly reduced in flies carrying *elav^3A^-GAL4>UAS-Caz-IR_1-167_* (*elav^3A^/Caz-IR_1-167_*) and those carrying *elav^3A^-GAL4>UAS-Caz-IR_363-399_* (*Caz-IR_363-399_/+;elav^3A^/+*) compared with its intensity in either driver control flies (*elav^3A^/+*) or responder control flies (*UAS-Caz-IR_1-167_/+*) (Figure 2B). There was a significant increase in Caz protein level in CNS extracts from the flies carrying *elav^3A^/Caz-IR_180-346_* compared with those from control flies carrying *elav^3A^/+* with unknown causes (Figure 2B). These results indicate that the anti-Caz antibody can specifically detect the Caz protein. These data also confirmed that *Caz* is effectively knocked down in flies carrying *elav^3A^/Caz-IR_1-167_* and *Caz-IR_363-399_/+;elav^3A^/+*, but it is not knocked down in flies carrying *elav^3A^-GAL4>UAS-Caz-IR_180-346_*, which we did not therefore use in the subsequent experiments.

### The Caz protein is localized in the larval and adult central nervous system of *Drosophila*

**Figure 3:**
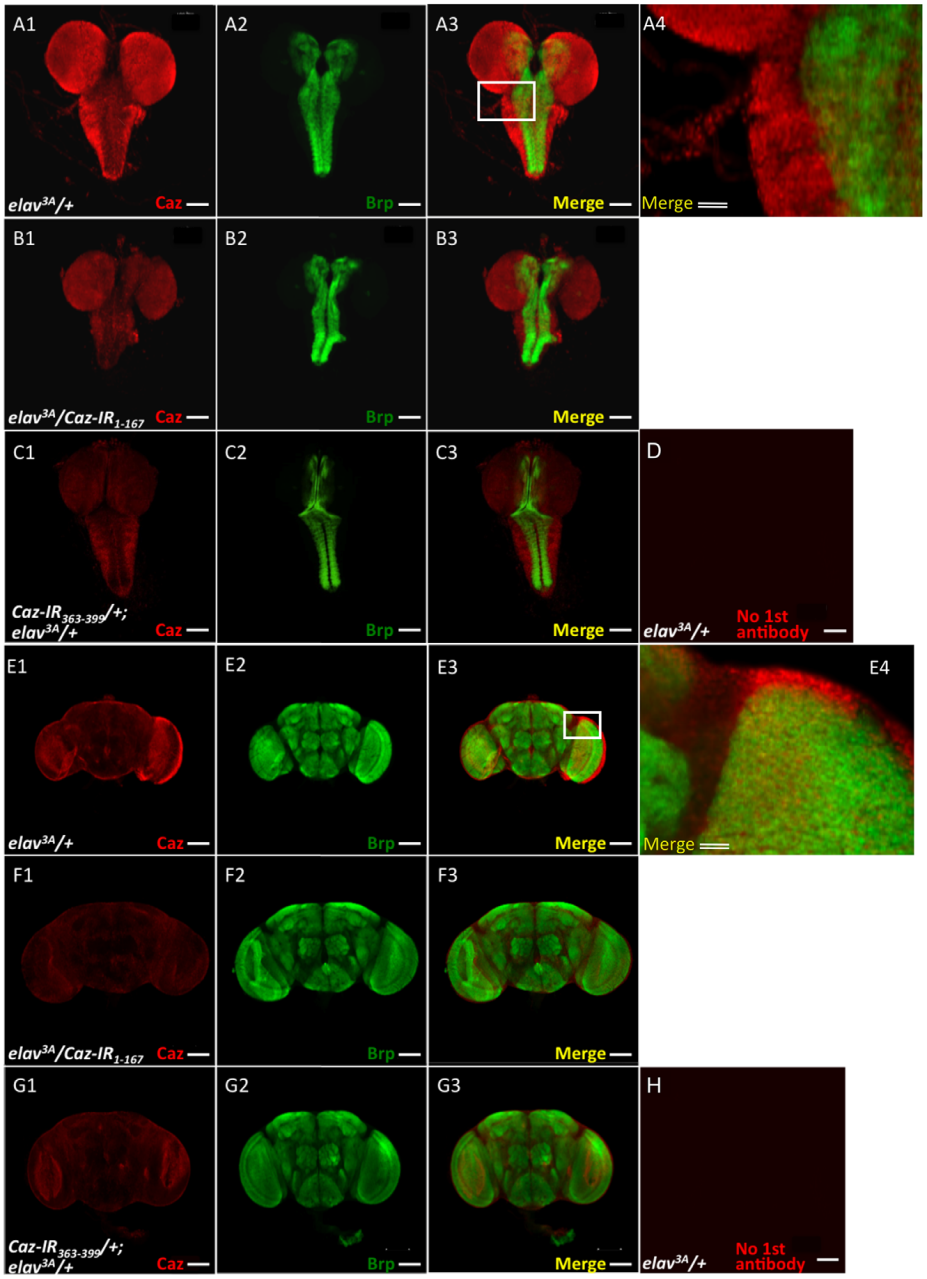
Immunohistochemical localization of Caz in larval and adult brains. Brain-ventral ganglia complexes from third instar larvae (A–D) and whole mount adult heads (E–H) were stained with the polyclonal anti-Caz antibody (A-1, B-1, C-1, E-1, F-1, G-1) or with an antibody against the neuropil marker Bruchpilot (Brp) (A-2, B-2, C-2, E-2, F-2, G-2). Merged confocal images of the two stains are shown at right (A-3, B-3, C-3, E-3, F-3 and G-3, respectively). Higher-magnification images of the boxed area in A-3 and E-3 are shown in A-4 and E-4, respectively. (D, H) Images of staining in the absence of first antibody. A-1 to A-4, E-1 to E-4, controls carrying *elav^3A^/+*; B-1 to B-3, F-1 to F-3, *Caz*-knockdown flies carrying *elav^3A^/Caz-IR_1-167_*; C-1 to C-3, G-1 to G-3, *Caz*-knockdown flies carrying *Caz-IR_363-399_/+;elav^3A^/+*. Caz antibody immunoreactivity decreased in CNS tissues from both the third instar larvae and the adult flies carrying *elav^3A^/Caz-IR_1-167_* (B-1, F-1) and *Caz-IR_363-399_/+;elav^3A^/+* (C-1, G-1). The single bars indicate 100 μm. The double bars indicate 20 μm.

The polyclonal anti-Caz antibody was used to examine the expression pattern of the Caz protein in the CNS of third instar *Drosophila* larvae and adult flies (Figure 3). *Drosophila* Caz was strongly expressed in the CNS of both larvae (Figure 3, A1) and adults (Figure 3, E1). No signal was generated in the absence of the primary anti-Caz antibody (Figure 3, D, H) indicating that this signal is specific for detection of the Caz protein. Moreover, the anti-Caz antibody signal did not overlap with the signal of the presynaptic marker Bruchpilot (Brp) that was detected using an anti-Brp antibody (Figure 3, A3-A4, E3-E4). This finding indicates that Caz localizes in a region other than synaptic areas both in third instar larvae and in adult flies (Figure 3, A1-A4, E1-E4), and suggests that Caz performs its physiological functions in neuronal cell bodies and/or their axons.

**Figure 4:**
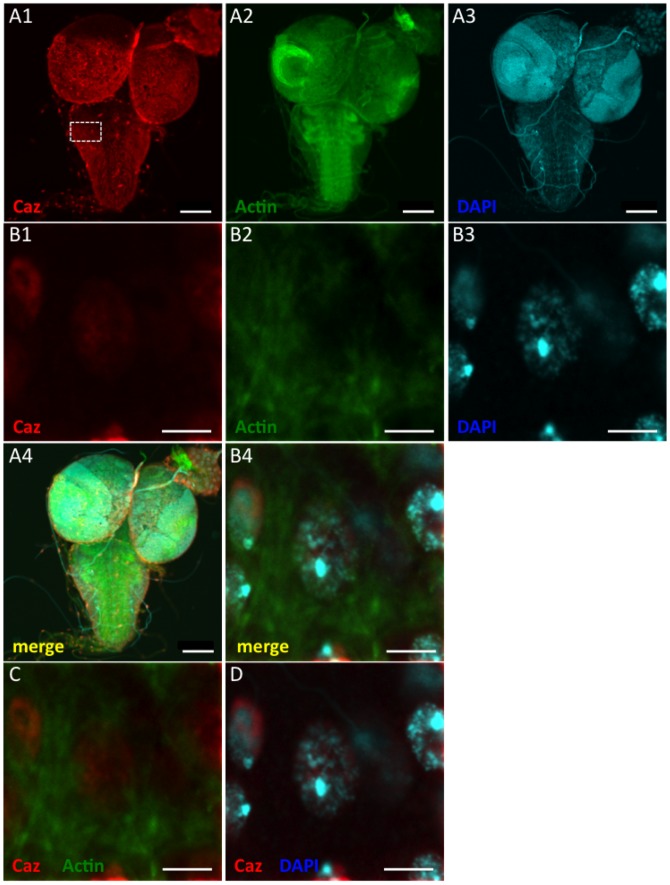
Intraneuronal localization of Caz in larval brains. Brain-ventral ganglia complexes from third instar larvae (A–D) were stained with the anti-Caz antibody (A-1, B-1), diamino-2-phenylidole (DAPI) (nuclear staining; A-2, B-2) or phalloidin (F-actin staining; A-3, B-3). Panels B-1 to B-4 are higher magnification images of the boxed area in A-1. Merged confocal images of A-1 to A-3, B-1 to B-3, B-1 and B-2, and B-1 and B-3 are shown in A-4, B-4, C, and D, respectively. The bar indicates 100 μm (A) or 5 μm (B–D). Anti-Caz antibody-immunoreactivity was detected in the nucleus of neuronal cells and did not co-localize with actin filaments, which stained with phalloidin. Since Caz did not co-localize with DAPI, which stains chromosomes, Caz must therefore localize in the nucleoplasm.

Regarding the precise localization of the Caz protein in neuronal cell bodies, Caz immunoreactivity was detected in the nucleus of neuronal cells of third instar larvae and did not co-localize with actin filaments, which are cytosolic proteins (Figure 4). However, within the nucleus, Caz did not co-localize with diamino-2-phenylidole (DAPI), suggesting that Caz is not localized on chromosomes but is localized in the nucleoplasm (Figure 4, C and D).

### Neuron-specific *Caz* knockdown causes fly mobility defects

To analyze the effect of *Caz* knockdown on fly phenotypes, we first investigated whether fly viability was affected by whole-body knockdown of *Caz*. Using an *Act5C-GAL4* driver that expresses GAL4 in the whole body of the fly, we analyzed the phenotypes of flies in which *Caz* double-stranded RNA was expressed throughout the whole body (Table 1). When crossed at 28°C, *UAS-Caz-IR_1-167_* was lethal at the pupal stage for all fly strains that carried it, while the strains carrying *UAS-Caz-IR_180-346_* were viable. When crossed at 25°C to decrease the expression levels of *Act5C-GAL4*, almost all of the strains carrying *UAS-Caz-IR_1-167,_* for which *UAS-Caz-IR_1-167_* had been lethal when crossed at 28°C, changed to be viable.

**Table 1:** Established fly strains carrying *UAS-Caz-IR*.

Transgene	Strain	Chromosome linkage	Act5C-GAL4>		elav^3A^-GAL4>
			28°C	25°C	
*UAS-Caz- IR_1-167_*	3	III	lethal	lethal	LD (+)
	4	III	lethal	NE	ND
	11	II	lethal	NE	ND
	21	III	lethal	NE	ND
*UAS-Caz- IR_180-346_*	11	II	NE	NE	NE
	12	II	NE	NE	NE
	17	II	NE	NE	ND
	22	III	NE	ND	LD (−)
	24	III	NE	NE	LD (−)
	32	III	ND	ND	ND
	33	III	ND	NE	ND
*UAS-Caz-IR_363-399_*		II	NE	ND	LD (+)

LD: locomotive dysfunction, NE: no effect, ND: not determined.

We next established transgenic fly lines in which *Caz* double-stranded RNA was specifically expressed in neuronal tissue by crossing the transgenic flies with the *elav^3A^-GAL4* line. As shown above in the immunoblotting analyses of the fly CNS (Figure 2), the expression levels of the Caz protein were much decreased in strain 3 of the fly lines that carried *elav^3A^/Caz-IR_1-167_* and in the fly line carrying *Caz-IR_363-399_/+;elav^3A^/+,* compared with the control flies. However, Caz expression levels did not show any detectable decreases in the fly lines carrying *elav^3A^-GAL4>UAS-Caz-IR_180-346_*. Similar to these results of immunoblotting analyses, immunostaining of the CNS of third instar larvae (Figure 3, B1, C1) and adult flies (Figure 3, F1, G1) showed that immunoreactivity detected with the anti-Caz antibody also decreased in the CNS tissues derived from the fly lines carrying *elav^3A^/Caz-IR_1-167_* (strain 3) (Figure 3, B1: larva, F1: adult fly) and *Caz-IR_363-399_/+;elav^3A^/+* (Figure 3, C1: larva, G1: adult fly). These results confirmed that *Caz* is effectively knocked down in the CNS of those two lines of transgenic flies.

**Figure 5:**
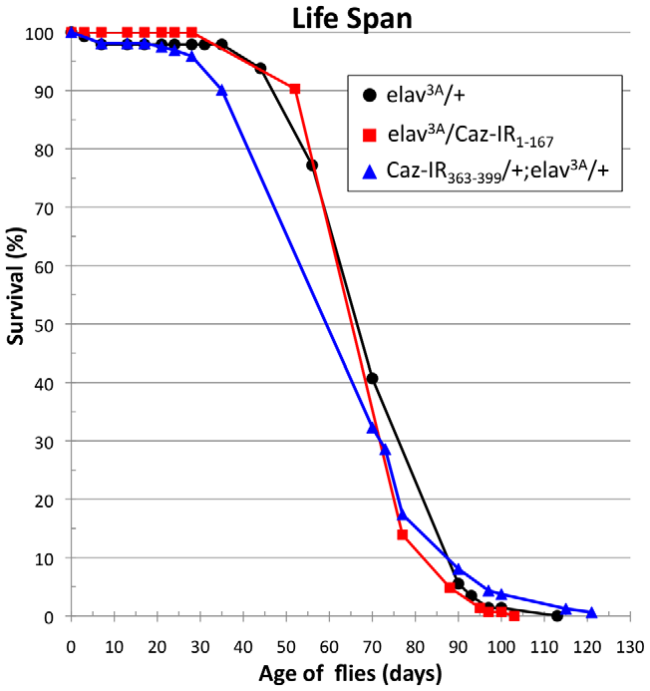
Life-span analyses of flies of each genotype. Percentage survival of adult male flies of the indicated genotypes is shown. Flies were collected from 20 different batches. The total number of flies counted was: *elav^3A^/+* (n = 145), *elav^3A^/Caz-IR_1-167_* (n = 144) and *Caz-IR_363-399_/+;elav^3A^/+* (n = 161). There were no significant differences in the life span of flies with the indicated genotypes.

To examine the effects of neuron-specific *Caz*-knockdown on the fly life span, we next determined the life span of each genotype (Figure 5). We examined adult flies until 120 days after eclosion, but there were no significant differences in life span between the control flies carrying *elav^3A^/+* (n = 145) and those carrying *elav^3A^/Caz-IR_1-167_* (n = 144) or *Caz-IR_363-399_/+;elav^3A^/+* (n = 161), in which the CNS expression of Caz was efficiently knocked down (Figure 5). The average life span of the control flies was 73.9 days, whereas flies carrying *elav^3A^/Caz-IR_1-167_* and *Caz-IR_363-399_/+;elav^3A^/+* lived an average of 76.5 days and 70.7 days, respectively. Fly life spans were therefore not significantly different between the control and neuron-specific *Caz*-knockdown flies.

**Figure 6:**
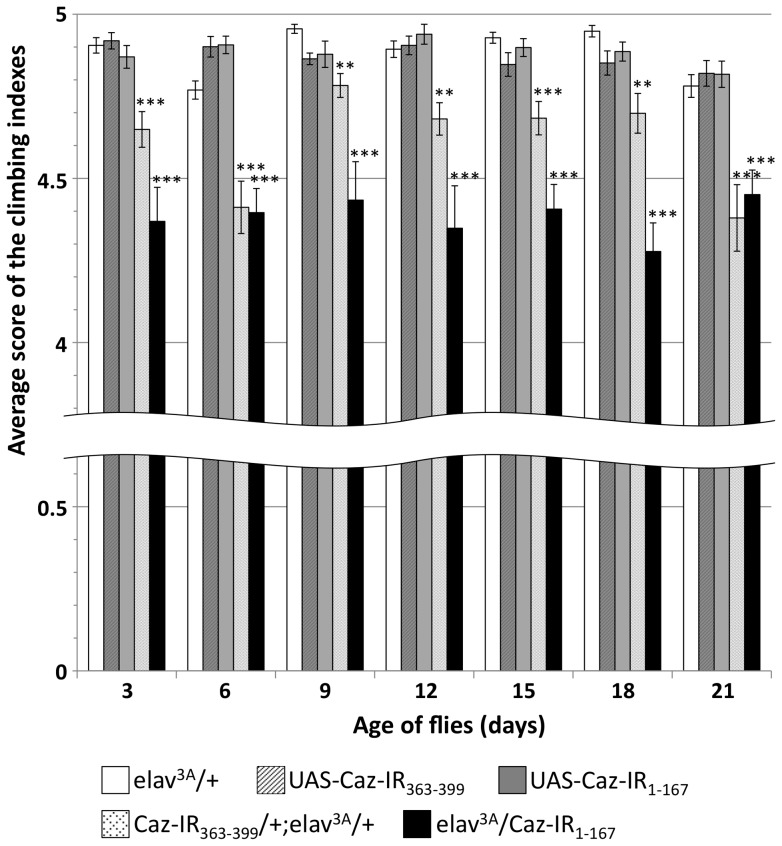
Climbing assays. Five independent tests were performed for each genotype. The total number of flies counted was: *elav^3A^/+* (a driver control, n = 309), *UAS-Caz-IR_363-399_/+* (a responder control, n = 222), *UAS-Caz-IR_1-167_/+* (a responder control, n = 246), *Caz-IR_363-399_/+;elav^3A^/+* (n = 265) and *elav^3A^/Caz-IR_1-167_* (n = 238). There was no significant difference in climbing abilities between the driver and responder control flies in each day after eclosion. Flies carrying *elav^3A^/Caz-IR_1-167_* or *Caz-IR_363-399_/+;elav^3A^/+* showed a significantly reduced ability to climb upwards compared to *elav^3A^/+* flies in each examined day. The horizontal bars indicate standard errors of mean values. ***p<0.001, **p<0.005.

In order to further evaluate the functional effects of neuron-specific *Caz* knockdown, we then performed climbing assays of the *Caz*-knockdown fly strains (Figure 6). The flies carrying *elav^3A^/Caz-IR_1-167_* showed reduced mobility both on day 3 (−10.7%) and day 21 (−9.3%) compared to the control flies carrying *elav^3A^/+*. Similarly *Caz-IR_363-399_/+;elav^3A^/+* carrying flies showed reduced mobility both on day 3 (−5.1%) and on day 21 (−10.6%). All of these reductions in mobility were statistically significant (p<0.001). These results indicate that *Caz* is involved in locomotion.

### Caz regulates the formation of motoneurons at presynaptic terminals in the NMJ

**Figure 7:**
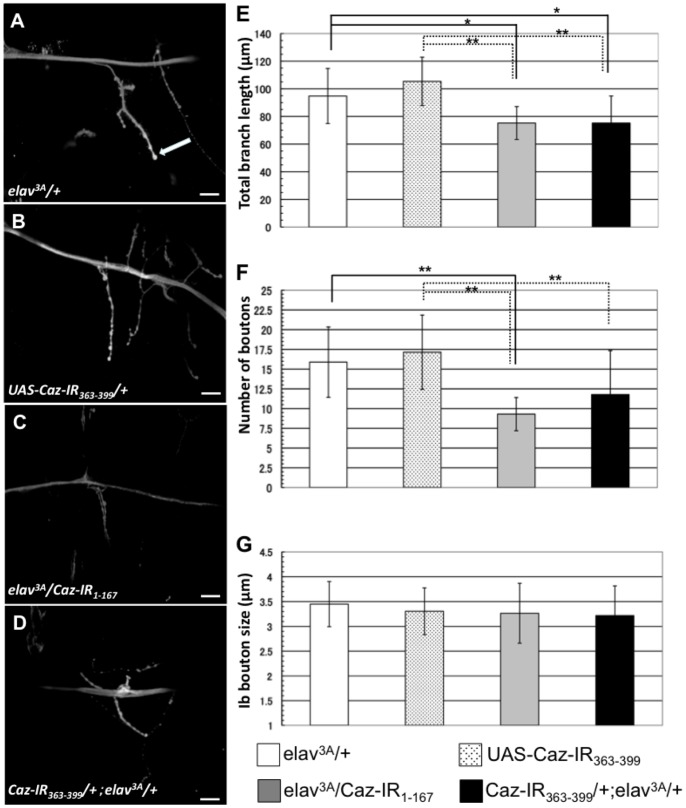
Confocal images of anti-HRP staining of muscle 4 synapses in third instar larvae. A representative image of the indicated genotypes is shown; (A) *elav^3A^/+* (a driver control), (B) *UAS-Caz-IR_363-399_/+* (a responder control), (C) *elav^3A^/Caz-IR_1-167_* and (D) *Caz-IR_363-399_/+;elav^3A^/+*. The bar indicates 20 μm. (E) Total branch length of the NMJ from muscle 4 for each of the indicated genotypes. n = 9 for each genotype. (F, G) The number (F) and the size (G) of the synaptic boutons for each of the indicated genotypes. (F) n = 9 for each genotype. (G) The size of Ib bouton (indicated with an arrow in A) was measured. n = 30 for *elav^3A^/+*, n = 34 for *Caz-IR_363-399_/+*, n = 27 for *elav^3A^/Caz-IR_1-_*_167_, n = 34 for *Caz-IR_363-399_/+elav^3A^/+.* The *Caz*-knockdown flies did not show any apparent changes in NMJ structure. However, the total length of synaptic branches of the motoneurons was significantly decreased in each Caz-knockdown fly strain (*elav^3A^/Caz-IR_1-167_* and *Caz-IR_363-399_/+;elav^3A^/+*) compared to the both driver and responder control flies (E). The flies carrying *elav^3A^/Caz-IR_1-167_* showed the significantly decreased number of the synaptic boutons compared to the both driver and responder control flies, and so did the flies carrying *Caz-IR_363-399_/+;elav^3A^/+* compared to the responder controls (F). There were no significant differences in the size of synaptic boutons among those 4 genotypes (G). The horizontal bars indicate standard errors of mean values. *p<0.05, **p<0.01.

Based on the fact that *Caz*-knockdown flies showed motor deficits in the climbing assays, together with the fact that *FUS*, the human counterpart of *Caz*, is involved in ALS that impairs motor neurons, we therefore decided to analyze the morphology of motoneuron presynaptic terminals at NMJs in these flies. Because most motoneurons of the adult fly originate from larval motoneurons, we compared the NMJ structure of the larvae of *elav^3A^/Caz-IR_1-167_* and *Caz-IR_363-399_/+;elav^3A^/+* flies with that of larvae of control flies carrying *elav^3A^/+* or *UAS-Caz-IR_363-399_/+.* None of these *Caz*-knockdown fly larvae showed apparent changes in NMJ structure (Figure 7, A-D). However, measurement of the total length of synaptic branches of motoneurons in these larvae indicated that the total branch length was significantly decreased in *elav^3A^/Caz-IR_1-167_* (75.3±11.9 μm) and *Caz-IR_363-399_/+;elav^3A^/+* (75.3±19.5 μm) flies compared to that of the both driver (*elav^3A^/+*; 94.8±19.9 μm) and responder (*UAS-Caz-IR_363-399_/+*; 105.4±17.5 μm) control *flies.* (Figure 7, E). The flies carrying *elav^3A^/Caz-IR_1-167_* showed the significantly decreased number of the synaptic boutons (9.3±2.1) compared to the both driver (15.9±4.5) and responder (17.1±4.7) control flies, and so did the flies carrying *Caz-IR_363-399_/+;elav^3A^/+* (11.8±5.6) compared to the responder controls (Figure 7, F). There were no significant differences in the size of synaptic boutons among those 4 genotypes (Figure 7, G). These results indicate that Caz is required for synaptic terminal growth at the NMJ.

## Discussion

We showed here that *Drosophila* Caz is strongly expressed in the central nervous system of larvae and adults. Caz did not colocalize with the presynaptic protein Brp, suggesting that Caz performs its physiological functions in neuronal cell bodies and/or their axons. In order to clarify whether or not disruption of the physiological functions of Caz are critical for the development of neurodegeneration even in the absence of abnormal Caz aggregates, we established fly models in which the *Caz* gene, which is the *Drosophila* FUS homologue, was knocked down. We demonstrated that neuron-specific knockdown of *Caz* did not affect the life span of the *Caz*-knockdown flies but did reduce the climbing abilities of adult flies, and also caused anatomical defects in presynaptic terminals of motoneurons in third instar larvae. These results suggested that a decrease in *Caz* expression is sufficient for the development of defects in locomotive abilities and for a decrease in the total length of synaptic branches of motoneurons at the NMJs in this *Drosophila* model. These data may indicate that the loss of physiological FUS functions in motoneurons would be more fundamental than the formation of cytoplasmic FUS aggregates in the pathogenesis of human FUS-related ALS/FTLD.

To eliminate the possibility that off-target effects of our RNAi construct that contained inverted repeats might generate the observed phenotypes, we used two different Caz inverted repeat constructs (*UAS-Caz-IR_1-167_* and *UAS-Caz-IR_363-399_*) whose target sequences did not overlap with each other. We established four transgenic fly strains carrying *UAS-Caz-IR_1-167_* as listed in Table 1. We also obtained a fly strain carrying *UAS-Caz-IR_363-399_* from the Vienna *Drosophila* RNAi center (VDRC). This fly strain carries an RNAi that is targeted to the region corresponding to residues 363-399 of *Drosophila* Caz (*UAS-Caz-IR_363-399_*), We then crossed these transgenic flies with the *elav^3A^-GAL4* line to specifically express *Caz* double stranded RNA in neuronal tissues. Each independent fly strain carrying *elav^3A^/Caz-IR_1-167_* showed essentially the same phenotype as the strain carrying *Caz-IR_363-399_/+;elav^3A^/+*. These results suggest that the phenotypes observed in the neuron-specific Caz-knockdown flies were not due to an off-target effect but rather to a reduction in Caz protein levels.

Mutations in the *FUS* gene are associated with inherited forms of both ALS and FTLD [15], [16], [18], [19]. The *FUS* gene was originally identified in a study that found that the FUS protein forms part of a fusion protein with the transcription factor CHOP, which arises due to a chromosomal translocation in liposarcoma [26]. It has been reported that there are both dominantly and recessively inherited families of ALS with *FUS* mutations [15]. Before the discovery of these *FUS* mutations in familial ALS, mutations in the *TARDBP* gene that encodes another RNA-binding protein, TDP-43, had been reported to be associated with familial ALS and FTLD [7]-[14]. Both the *FUS* gene and the *TARDBP* gene encode an RNA-binding protein equipped with an RRM, and should therefore be involved in RNA processing, splicing, and RNA metabolism. Since FUS and TDP-43 have substantial similarities in their protein structure and putative functions, they could therefore cause ALS or FTLD through common pathogenic processes [2], [38]. However, the mechanisms through which mutations in *FUS* or *TARDBP* cause ALS and FTLD are not known, and both toxic gain-of-function and loss-of-function models have been proposed [2], [38]. ALS-associated mutant forms of TDP-43 and FUS are known to form abnormal cytosolic aggregates [15], [16], [35], [39]–[41], and high-level overexpression of either wild-type or mutant TDP-43 is neurotoxic in mice, zebra fish and *Drosophila* [42]–[47]. One recent study reported that a *Drosophila* model in which targeted expression of mutant human FUS in *Drosophila* motor neurons led to locomotor dysfunction [48]. These findings would support the toxic gain-of-function model. However, overexpression of mutant proteins may also perturb the activity of endogenous TDP-43, supporting the loss-of-function model [49]. Similarly, the targeted expression model mentioned above reported that deletion of the nuclear export signal rescued toxicity associated with mutant FUS, suggesting that delocalization of FUS from the nucleus to the cytoplasm, namely the loss-of-nuclear-function, would be necessary for neurodegeneration [48]. In this study, we demonstrated that neuron-specific knockdown of *Caz*, the *Drosophila FUS* homologue, could induce a defect in fly locomotive abilities as well as degeneration of motoneurons at NMJs in the model flies. There has been one previous report that showed that flies lacking TBPH, the *Drosophila* TDP-43 homologue, present deficient locomotive behaviors, reduced life span and anatomical defects at the NMJs [37]. Regarding *FUS* and its homologues, one recent study reported that *Drosophila* mutants in which the *Caz* gene was disrupted exhibited decreased adult viability, diminished locomotor speed and reduced life span compared with controls, and that these phenotypes were fully rescued by wild-type human *FUS*, but not by ALS-associated mutant *FUS* [50]. These reports, together with our results, demonstrated that a lack of physiological functions of FUS or TDP-43 in the nucleus is sufficient for induction of locomotive dysfunction and motoneuron degeneration, which recapitulate the phenotypes of ALS, and they therefore imply that the loss of physiological FUS functions are sufficient for the development of pathogenic processes similar to those that occur in FUS- or TDP-43-related ALS/FTLD, in the absence of cytosolic aggregates that may be toxic to motoneurons in ALS/FTLD.

There have been a few previous studies in which loss-of-function animal models of FUS-related human disorders were generated. FUS knockout mice show perinatal lethality and defects in B lymphocyte development [51]. Additionally, the hippocampal pyramidal neurons of these FUS-null mice exhibited abnormal spine morphology and lower spine density [52]. One report showed that surviving knockout mice exhibited male sterility [53]. However, the neurodegenerative phenotypes of these mice have not been reported to date. With regard to *Drosophila* models, one recent paper that was mentioned above presented a mutant fly strain (named the *Caz1* mutant) in which 58% of the *Caz* gene was deleted by creating a small genomic deletion [50]. This fly model developed a phenotype of disturbed locomotion that is similar to that observed in the *Caz*-knockdown flies in the present study. The differences between the *Caz1* mutant and our fly models were as follows; 1) the *Caz1* mutant did not show any morphological abnormalities at the NMJs i.e., shortening of the presynaptic terminals of motoneurons and decrease in the number of synaptic boutons, both of which were observed in our *Caz*-knockdown models. 2) The *Caz1* mutant showed reduced life spans, which were not observed in our models, and this life-span defect of *Caz1* mutant could be fully rescued by expression of wild-type fly *Caz* or wild-type human *FUS* in neurons using *elav-Gal4*. The difference in life span between the *Caz1* mutant and our *Caz*-knockdown models might be caused by differences in the expression pattern of the transgenes between the two models; in our fly models *Caz* gene expression was knocked down specifically in the nervous system, whereas, in the short-lived *Caz1* mutant flies, *Caz* was disrupted throughout the whole body. In our *Caz*-knockdown models, the expression of Caz protein was knocked down to 40–60% in the CNS (Figure 2), but their life spans were not reduced. Together with the fact that the reduced life span of the *Caz1* mutant was rescued by the neuronal expression of wild-type *Caz*, our results suggest that substantial expression of *Caz* in neuronal tissues, even though it is not fully expressed, could sufficiently keep their life spans within normal range. Our model flies also demonstrated that normal expression of *Caz* in neurons is essential for the elongation of synaptic branches of motoneurons at NMJs, and therefore that *Caz*-knockdown would induce impaired maturation of these synaptic branches, resulting in the observed locomotive deficit in our model flies, in the absence of any non-neuronal effect of the Caz protein.

In conclusion, we established fly models with neuron-specific knockdown of the *Drosophila* FUS homologue, and showed that those flies developed locomotive deficits as well as anatomical defects of motoneurons at NMJs. Our results indicate that the loss of physiological FUS functions in the nucleus is more likely to be the fundamental pathogenic mechanism that causes FUS-related ALS/FTLD than the toxicity of cytoplasmic aggregates. These data further indicate future research directions, suggesting that it will be necessary to identify target molecules, including nuclear proteins and/or RNA species that associate with FUS, in order to elucidate the molecular mechanisms leading to neuronal dysfunction in FUS-associated ALS/FTLD and to develop the disease-modifying therapies that are eagerly desired in those relentless neurodegenerative diseases. In any event, the *Drosophila* model that we established in the present study, which recapitulates key features of human ALS, would be suitable for the screening of genes and chemicals that can modify these pathogenic processes that lead to the degeneration of motoneurons in ALS.

## Materials and Methods

### Fly stocks

Fly stocks were maintained at 25°C on standard food containing 0.7% agar, 5% glucose and 7% dry yeast. Canton S was used as the wild type. W; *UAS-Caz-IR*; + (CG3606) was obtained from the Vienna *Drosophila* RNAi center (VDRC). The RNAi of this strain was targeted to the region corresponding to residues 363-399 of *Drosophila* Caz (*UAS-Caz-IR_363-399_*). P{*GAL4-elav.L*}3A (elav^3A^-GAL4) was provided by Dr. Bryan Stewart [54]. The Act5C-GAL4 strain was obtained from the Bloomington *Drosophila* stock center. Establishment of the lines carrying GMR-GAL4 was as described previously [55].

### Comparison of amino acid sequences of human FUS and *Drosophila* Caz

The amino acid sequence of *Drosophila* Caz was retrieved from the Flybase (http://flybase.org). The identity and the similarity of *Drosophila* Caz and human FUS were compared using BLAST (http://blast.genome.jp/) and FASTA (http://fasta.genome.jp/). FASTA was used for comparison of the entire sequences, and BLAST was used for comparison of each corresponding domain between Caz and FUS.

### Establishment of the transgenic flies

To establish transgenic fly lines carrying *UAS-Caz-IR*, 500-bp fragments of Caz ORFs (*UAS-Caz-IR_1-167_*; 5′-ATGGAACGTGGCGGTTATGGTGGT to 5′-AGAACAAGGAGACCGGCGC, *UAS-Caz-IR_180-346_*; 5′-ATGCTGCACAATCCGCCATTGAAT to 5′-CAACAGAGATCGCGGTGGC) from *Caz* cDNA clone CG3606 were amplified, and then individually cloned into the pENTR/D-TOPO vector (Invitrogen Life Technologies Japan Corporation, Tokyo, Japan), in which each trigger sequence of *Caz* was placed between the *attL1* and *attL2* recombination sequences. Following confirmation by sequencing, two copies of each trigger sequence were transferred into the pRISE transformation vector that contains a characteristic inverted repeat of *attR1-cm^r^-ccdB-attR2* in a recombination cassette by an *in vitro* reaction mediated by LR Clonase (Invitrogen), a DNA recombinase that specifically recognizes the *attL* and *attR2* target sites [56]. Due to the recombination reaction between the *attL* and *attR2* sites, the ccdB sequence was replaced by the target cDNA, resulting in the cloning of a head-to-head inverted repeat (IR) of Caz into the plasmid.

These plasmids were verified by sequencing and then injected into embryos to obtain stable transformant lines carrying *UAS-Caz-IR*. P element-mediated germ line transformation was carried out as described previously [57], and F1 transformants were selected on the basis of white-eye color rescue [58]. Four and seven transgenic strains carrying *UAS-Caz-IR_1-167_* and *UAS-Caz-IR_180-346_* were established, respectively (responder controls), as listed in Table 1. To drive expression of *Caz* double stranded RNA in the whole body of the flies or specifically in neuronal tissues, we crossed the transgenic flies with either the *Act5C-GAL4* line or the *elav^3A^-GAL4* line (*elav^3A^-GAL4/+*; a driver control). Each transgenic strain showed a consistent phenotype (Table 1).

### Production of rabbit anti-Caz antibodies

Rabbit anti-Caz antibodies were produced by MEDICAL & BIOLOGICAL LABORATORIES Co., Ltd (MBL, Ina, Japan). The peptides, N-NKTGNYEPPPNYGKQGC-C (residues 29–45; the underlined C residue C was an added residue) and N-CRDGGPMRNDGGMRSRPY-C (residues 383–399), which correspond respectively to the N- and C-terminal sequences of Caz, were individually conjugated with KLH (Keyhole limpet hemocyanin). These two KLH-conjugated peptides were mixed with Freund's complete adjuvant to provide a suspension, which was injected intradermally into rabbits (Female Japanese White). The rabbits were then boosted with inoculations of an immunogen of the same quality once a week for 7 weeks, and a terminal bleed was performed to collect the maximum amount of serum. The serum was purified by affinity chromatography against the synthesized peptides using a Protein G column.

### Immunoblotting analysis

Protein extracts from the central nervous system (CNS) of *Drosophila* carrying *elav^3A^-GAL4/+, UAS-Caz-IR_180-346_/+*, *UAS-Caz-IR_1-167_/+* and *elav^3A^-GAL4>UAS-Caz-IR* were prepared as previously described [59]. Briefly, the CNS was excised from third instar larvae and homogenized in a sample buffer containing 50 mM Tris-HCl (pH 6.8), 2% SDS, 10% glycerol, 0.1% bromophenol blue and 1.2% β-mercaptoethanol. The homogenates were boiled at 100°C for 5 min, and then centrifuged. The supernatants (extracts) were electrophoretically separated on SDS-polyacrylamide gels containing 12% acrylamide and then transferred to polyvinylidene difluoride (PVDF) membranes (Bio-Rad, Osaka, Japan). The blotted membranes were blocked with TBS/0.05% Tween containing 5% skim milk for 1 h at 25°C, followed by incubation with rabbit polyclonal anti-Caz at a 1∶5,000 dilution for 16 h at 4°C. After washing, the membranes were incubated with HRP-conjugated anti-rabbit IgG (GE Healthcare Bioscience, Tokyo, Japan) at 1∶10,000 dilution for 2 h at 25°C. Antibody binding was detected using ECL Western blotting detection reagents (GE Healthcare Bioscience) and images were analyzed using a Lumivision Pro HSII image analyzer (Aisin Seiki, Kariya, Japan). To ensure equal protein loading in each lane, the membranes were also probed with an anti-α-tubulin antibody after stripping the complex of anti-Caz antibody and HRP-conjugated anti-rabbit IgG. For the detection of α-tubulin, mouse anti-α-tubulin monoclonal antibody (1∶5,000 dilution, Sigma, Tokyo, Japan) and an HRP-conjugated anti-mouse IgG (1∶10,000 dilution, GE Healthcare Bioscience) were used as the primary and secondary antibodies, respectively.

### Immunostaining

For immunohistochemical analysis, CNS tissues of third instar larvae and adult flies were dissected, and fixed in 4% paraformaldehyde/PBS for 15 min at 25°C. After washing with PBS containing 0.3% Triton X-100, the samples were blocked with blocking buffer (PBS containing 0.15% Triton X-100 and 10% normal goat serum) for 30 min at 25°C, and then incubated with diluted primary antibodies in the blocking buffer for 20 h at 4°C. The following antibodies were used; 1∶1,000 diluted rabbit anti-Caz antibody and 1∶100 diluted mouse anti-Brp antibody (Developmental Studies Hybridoma Bank [DSBH] nc82). After extensive washing with PBS containing 0.3% Triton X-100, samples were incubated with secondary antibodies labeled with either Alexa 546 or Alexa 488 (1∶400; Invitrogen) diluted in the blocking buffer, in the dark, for 2 h at 25°C. After extensive washing with PBS containing 0.3% Triton X-100 and PBS, samples were mounted in Fluoroguard Antifade Reagent (Bio-Rad) and observed under a Zeiss LSM510 confocal laser scanning microscope.

For NMJ staining, third instar larvae were dissected in HL3 saline [60] and fixed in 4% paraformaldehyde/PBS for 30 min. The blocking buffer was 2% bovine serum albumin (BSA)/PBS/0.1% TritonX-100. FITC-conjugated goat anti-HRP (1∶1,000, MP Biochemicals) was used as the detection antibody. The samples were mounted and observed under a Zeiss LSM510 confocal laser scanning microscope. MN4 (Ib) in muscle 4 in abdominal segment 2 was quantified. Images were acquired using a Zeiss LSM510 by merging 1 μm interval z-sections onto a single plane. Nerve terminal branch lengths were measured using Image J software.

To determine whether Caz is present in the nucleus or not, CNS tissues of third instar larvae were dissected, and fixed in 4% paraformaldehyde/PBS for 15 min at 25°C. After washing with PBS containing 0.3% Triton X-100, the samples were incubated with Alexa 488-conjugated phalloidin (1 unit/200 μl) in PBS containing 0.3% Triton X-100 for 20 min at 25°C. The samples were then blocked and reacted with the primary and the secondary antibodies as described for the immunohistochemical analysis described above, except that the mouse anti-Brp antibody was not used. After extensive washing with PBS containing 0.3% Triton X-100, the samples were stained with DAPI (0.5 μg/ml)/PBS/0.1% Triton X-100. Following washing with PBS containing 0.1% Triton X-100 and PBS, the samples were mounted and observed under a confocal laser scanning microscope (OLYMPUS Fluoview FV10i).

### Longevity assay

Longevity assays were carried out in a humidified, temperature controlled incubator at 25°C and 60% humidity on a 12-h light and 12-h dark cycle on standard fly food. Flies carrying *elav^3A^-GAL4/+* and *elav^3A^-GAL4>UAS-Caz-IR* were placed at 28°C, and newly eclosed adult flies were separated and placed in vials at a low density (10–20 flies per vial) with a male: female ratio of 1: 1. Every 3 days, they were transferred to new tubes containing fresh food and deaths were scored. Survival rate was determined by plotting a graph of the percentage of surviving flies versus days.

### Climbing assay

Climbing assays were performed as described previously [61]. Flies carrying *elav^3A^-GAL4/+*, *UAS-Caz-IR_363-399_/+*, *UAS-Caz-IR_1-167_/+*, and *elav^3A^-GAL4>UAS-Caz-IR* were placed at 28°C, and newly eclosed adult flies were separated and placed in vials at a density of 30 flies per vial (15 males and 15 females). Flies were transferred, without anesthesia, to a conical tube. The tube was tapped to collect the flies to the bottom, and they were then given 30 s to climb the wall. After 30 s the flies were collected at the bottom by tapping of the tube, and were again allowed to climb for 30 s. Similar procedures, all of which were videotaped, were repeated five times in total. For all of the climbing experiments, the height to which each fly climbed was scored as follows (score (height climbed)); 0 (less than 2 cm), 1 (between 2 and 3.9 cm), 2 (between 4 and 5.9 cm), 3 (between 6 and 7.9 cm), 4 (between 8 and 9.9 cm) and 5 (greater than 10 cm). The climbing index of each fly strain was calculated as follows; the sum of the products of each score multiplied by the number of flies for which that score was recorded, was calculated, and this number was then divided by five times the total number of flies examined. These climbing assays were carried out every 3 days until the 18th day after eclosion.

### Data analysis

All statistical analyses were performed using Microsoft Excel. The Mann-Whiney test was used for assessment of the statistical significance of comparisons between groups of data concerning median life span. For other assays the two-way ANOVA was used to determine the statistical significance of comparisons between groups of data. When the two-way ANOVA showed significant variation among the groups, Dunnet's test was subsequently used for pairwise comparisons of groups. All data are shown as means ± SEM.
